# The development of the BK-SE36 malaria vaccine candidate

**DOI:** 10.1186/1475-2875-13-S1-P67

**Published:** 2014-09-22

**Authors:** Nirianne MQ Palacpac, Masanori Yagi, Edward Ntege, Betty Balikagala, Adoke Yeka, Hiroki Shirai, Nahoko Suzuki, Takuya Okada, Bernard Kanoi, Arisue Nobuko, Takahiro Tougan, Ken J Ishii, Thomas G Egwang, Toshihiro Horii

**Affiliations:** 1 Research Institute for Microbial Diseases, Osaka University, Suita, Osaka, Japan; 2 Med Biotech Laboratories, Kampala, Uganda; 3 Makerere University School of Public Health, Kampala, Uganda; 4 The Research Foundation for Microbial Diseases of Osaka University, Kanonji, Kagawa, Japan; 5 National Institute of Biomedical Innovation, Ibaraki, Osaka, Japan

## 

Complex and widespread with about 90% of the global burden in sub-Saharan Africa, malaria remains a significant health problem. Malaria vaccines have the chance to make significant inroads, with RTS,S vaccine candidate as the most advanced candidate. However, the need for a highly efficacious, long-lasting vaccine is still unmet. Continued support for a repertoire of tools for *Plasmodium falciparum* is needed. A blood-stage vaccine is desirable to deal with morbidity and mortality secondary to *P. falciparum* infection, and thus would be a valuable strategy in the fight against malaria.

The *P. falciparum* serine repeat antigen-5 formulated with aluminum hydroxyl gel (BK-SE36) is a blood-stage malaria vaccine candidate. The processing of SERA5 is believed to mediate parasite egress from the infected red blood cell. Field studies show good correlations of anti-SERA antibodies to malaria protection against blood parasitemia, malaria symptoms and severe disease. A Phase 1a trial demonstrated its safety and immunogenicity in malaria naïve Japanese adults. A Phase 1b trial was conducted in the malaria endemic area of Northern Uganda to assess the vaccine’s safety and immunogenicity. BK-SE36 had an acceptable safety and immunogenicity profile. Post-trial, Stage 2 subjects were age-matched to 50 control individuals to compare malaria episodes 130–365 days post-second vaccination. Log-rank test reveal significant differences in BK-SE36 vaccinees vs control group in time-to-first episodes with parasitemias ≥5,000/μL and those with ≥5,000/μL + fever (protective efficacy for the latter is 72%, *P* < 0.01). BK-SE36 vaccinees have fewer multiple malaria episodes with high parasitemia. We also observed that the antibody titers against SE36 were boosted after episodes of natural infection. Above results show that BK-SE36 could provide some protection against natural *P. falciparum* infection until 1 year post-second vaccination.

